# Utilizing Machine Learning to Predict Neurological Injury in Venovenous Extracorporeal Membrane Oxygenation Patients: An Extracorporeal Life Support Organization Registry Analysis

**DOI:** 10.21203/rs.3.rs-3779429/v1

**Published:** 2023-12-22

**Authors:** Andrew Kalra, Preetham Bachina, Benjamin L. Shou, Jaeho Hwang, Meylakh Barshay, Shreyas Kulkarni, Isaac Sears, Carsten Eickhoff, Christian A. Bermudez, Daniel Brodie, Corey E. Ventetuolo, Glenn J. R. Whitman, Adeel Abbasi, Sung-Min Cho

**Affiliations:** Johns Hopkins University School of Medicine; Johns Hopkins University School of Medicine; Johns Hopkins University School of Medicine; Johns Hopkins University School of Medicine; Warren Alpert Medical School of Brown University; Warren Alpert Medical School of Brown University; Warren Alpert Medical School of Brown University; Faculty of Medicine, University of Tübingen; Perelman School of Medicine at the University of Pennsylvania, Philadelphia; Johns Hopkins University School of Medicine; Warren Alpert Medical School of Brown University; Johns Hopkins University School of Medicine; Warren Alpert Medical School of Brown University; Johns Hopkins University School of Medicine

**Keywords:** venovenous extracorporeal membrane oxygenation, machine learning, acute brain injury, neurological complications

## Abstract

**Background:**

Venovenous extracorporeal membrane oxygenation (VV-ECMO) is associated with acute brain injury (ABI), including central nervous system (CNS) ischemia (defined as ischemic stroke or hypoxic-ischemic brain injury) and intracranial hemorrhage (ICH). There is limited data on prediction models for ABI and neurological outcomes in VV-ECMO.

**Research Question:**

Can machine learning (ML) accurately predict ABI and identify modifiable factors of ABI in VV-ECMO?

**Study Design and Methods:**

We analyzed adult (≥18 years) VV-ECMO patients in the Extracorporeal Life Support Organization Registry (2009–2021) from 676 centers. ABI was defined as CNS ischemia, ICH, brain death, and seizures. Overall, 65 total variables were extracted including clinical characteristics and pre-ECMO and on-ECMO variables. Random Forest, CatBoost, LightGBM, and XGBoost ML algorithms (10-fold leave-one-out cross-validation) were used to predict ABI. Feature Importance Scores were used to pinpoint variables most important for predicting ABI.

**Results:**

Of 37,473 VV-ECMO patients (median age=48.1 years, 63% male), 2,644 (7.1%) experienced ABI: 610 (2%) and 1,591 (4%) experienced CNS ischemia and ICH, respectively. The median ECMO duration was 10 days (interquartile range=5–20 days). The area under the receiver-operating characteristics curves to predict ABI, CNS ischemia, and ICH were 0.67, 0.63, and 0.70, respectively. The accuracy, positive predictive, and negative predictive values for ABI were 79%, 15%, and 95%, respectively. ML identified pre-ECMO cardiac arrest as the most important risk factor for ABI while ECMO duration and bridge to transplantation as an indication for ECMO were associated with lower risk of ABI.

**Interpretation:**

This is the first study to use machine learning to predict ABI in a large cohort of VV-ECMO patients. Performance was sub-optimal due to the low reported prevalence of ABI with lack of standardization of neuromonitoring/imaging protocols and data granularity in the ELSO Registry. Standardized neurological monitoring and imaging protocols may improve machine learning performance to predict ABI.

## Introduction

Venovenous extracorporeal membrane oxygenation (VV-ECMO) is a mechanical circulatory support method for respiratory failure patients.^1^ VV-ECMO is associated with acute brain injury (ABI) including central nervous system (CNS) ischemia (ischemic stroke and hypoxic-ischemic brain injury (HIBI)) and intracranial hemorrhage (ICH). As ischemic strokes and ICH double the risk of mortality in VV-ECMO patients,^2^ determining risk factors for their occurrence may help us in minimizing their rate of occurrence. Prior analyses of the Extracorporeal Life Support Organization (ELSO) Registry, the largest international database of ECMO patients with over 200,000 cases using logistic regression showed that acidosis, hypoxemia, and coagulation disturbances immediately before cannulation were independently associated with ABI in VV-ECMO patients.^2^

Machine learning (ML) may be able to ascertain modifiable risk factors associated with ABI that may have been undetected in prior multivariable logistic regression models by unveiling relationships not visible using traditional regression.^3,4^ Furthermore, the ELSO Registry provides a theoretical advantage for using ML to predict ABI due its large sample size across many ECMO centers worldwide. A previous ELSO Registry study (n = 23,182) with VA-ECMO patients using ML to predict in-hospital mortality demonstrated good performance (area under the receiver-operating characteristic curve, AUC-ROC: 0.80).^5^ However, to date, there is a dearth of studies aimed at predicting neurological outcomes, such as ABI, in VV-ECMO patients using ML. Herein, we aimed to use ML to predict ABI and to identify associated risk factors using the largest ELSO database of adult VV-ECMO patients.

## Study Design and Methods

### Study design and population

This retrospective study was approved by the Johns Hopkins Hospital Institutional Review Board with a waiver of informed consent (IRB00216321). The ELSO Registry is an international multicenter registry collecting data from 676 ECMO centers (2009–2021) across the world.^6^ The Registry collects demographic information, baseline comorbidities, hemodynamic and arterial blood gas (ABG) information before and during ECMO support, neurological and systemic on-ECMO complications, and clinical outcomes including in-hospital mortality.^7^ *International Classification of Diseases, 10*^*th*^ *Revision (ICD-10)* codes were used to identify comorbidities.

#### Inclusion criteria:

Patients who were 1) 18 years of age or older; and 2) supported with VV-ECMO from 2009–2021 were included. An exploratory analysis on “Conversion” ECMO patients (those who were converted from venoarterial (VA)-ECMO to VV-ECMO or VV-ECMO to VA-ECMO) was included and performed.

#### Exclusion criteria:

Repeat ECMO runs within the same patient were excluded. Patients on VA-ECMO support were excluded.

### Data collection

**Figure 1:**
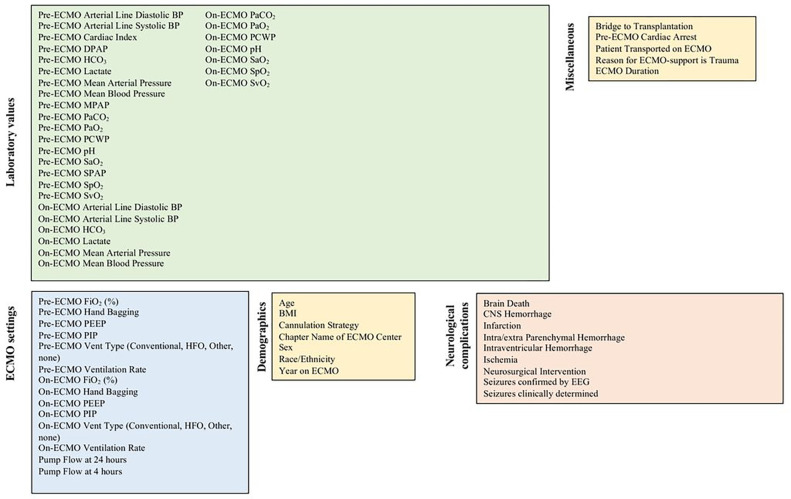
Variables used in our machine learning pipeline to predict ABI, ranging from demographics to on-ECMO laboratory values.

Figure 1 depicts all features collected for the ML pipeline(65 total). The ELSO Registry gathers ABG and hemodynamic information before and after ECMO cannulation, which were defined as “pre-ECMO” and “on-ECMO” variables. Within 6 hours of ECMO cannulation, pre-ECMO ABGs were drawn and tell pre-ECMO ventilator settings were recorded. The pre-ECMO ABG that was closest to the start of ECMO cannulation was used if multiple ABGs within the 6-hour period were present. The on-ECMO ABGs and hemodynamic variables nearest to 24 hours, determined 18–30 hours post cannulation, were used as post cannulation values. A trained ELSO data manager from each ELSO center abstracted data points that were meant to be collected concurrently such as oxygen saturations measured by pulse oximetry versus arterial blood gas. All pre-ECLS support codes (including cardiac arrest, mechanical cardiac support, vasopressor and inotrope infusions, bridge to transplant as an ECMO indication, patient transported to another ELSO center) represented conditions present within 24 hours of ECMO initiation.

### Definitions

ABI includes CNS ischemia, which was defined as infarction (ischemic stroke) and/or diffuse ischemia (HIBI), intra/extra parenchymal hemorrhage, intraventricular hemorrhage, seizures determined by electroencephalograph or clinically, and/or neurosurgical intervention (e.g., intracranial pressure monitor, external ventricular drain, or craniotomy). Ischemic stroke was determined by computed tomography (CT), ultrasound, or magnetic resonance imaging. HIBI was determined by CT. ICH was determined by CT and was defined as intra/extra parenchymal hemorrhage and/or intraventricular hemorrhage. The definitions for demographics, pre-ECMO support, hemodynamics, ABGs, and systemic complications are in the **Supplemental Methods.**

### Outcomes

The primary outcome was ABI while on ECMO support. Secondary outcomes were CNS ischemia and ICH (subgroups of ABI).

### Statistical analysis

Continuous variables were denoted as median and interquartile range (IQR). Categorical variables were portrayed as frequencies. Both the Wilcoxon rank-sum and Pearson’s chi-square tests were used to compare continuous and categorical variables, respectively. Statistical significance was considered P <0.05.

#### Data pre-processing

Categorical features were one hot-encoded before running ML algorithms. The default imputation method, multiple imputation, for each algorithm for Python packages such as XGBoost and CatBoost was used for missing data (maximum <30% missing data). All missing data are conveyed in **Supplemental Table 1**.

#### Machine Learning Algorithm and Pipeline

We analyzed the appropriateness of 4 different ML algorithms to predict ABI from the ELSO Registry, including Random Forest, CatBoost, LightGBM, and XGBoost. A Bayesian optimization was used to split the dataset at random into training (70%) and test (30%) sets. Hyperparameters were fine-tuned for all 4 algorithms. Using the fine-tuned hyperparameters, each of the 4 ML models was fitted onto the training set and then assessed on the test set with the top-performing model chosen for further optimization. We used random oversampling of patients with ABI in the training set at varying occurrences. For each oversampling frequency, we evaluated the model with a 10-cross-validation (CV) approach. Once we identified the most ideal oversampling rate, we used the most optimal performing model on the entire cohort using a leave-one-out-cross-validation (LOOCV) method. This method functions by using all observations in the training set except an individual (n=1) observation that is reserved to be used in the test set. This LOOCV stepwise technique was repeated across the entire cohort. Each observation was utilized as the test set. This method correspondingly produced “*N”* number of models that were subsequently trained and then tested on the holdout “*N”* observations. In the end, all observations were pooled to yield a combined “*N”* number of observations. We ensured the reproducibility of our results by using this LOOCV approach as it lessens the risk of bias by testing the ML algorithm on the whole cohort. We then computed the AUC-ROC, the area under the precision recall curve, and Brier scores to measure the predictive performance of each model. We also chose a cutoff that optimized the F1 score. Then, we calculated corresponding sensitivities, specificities, negative predictive values (NPV), and positive predictive values (PPV).

#### Important features in ML algorithms

To unveil a “black box” method in ML, we examined which variables were most essential to correctly predict ABI through Feature Importance Scores and Shapley Additive Explanations (SHAP) values. We analyzed the hierarchical feature importance in each model, which depicts the contribution of each feature in the boosted decision trees. Adescription of the interpretation of SHAP values is in the **Supplemental Methods.** Feature Importance Scores and SHAP values allowed us to identify the most relevant clinical features associated with each outcome. All statistical analyses were conducted using R Studio (R 4.1.2, www.r-project.org) and Python.

## Results

**Figure 2:**
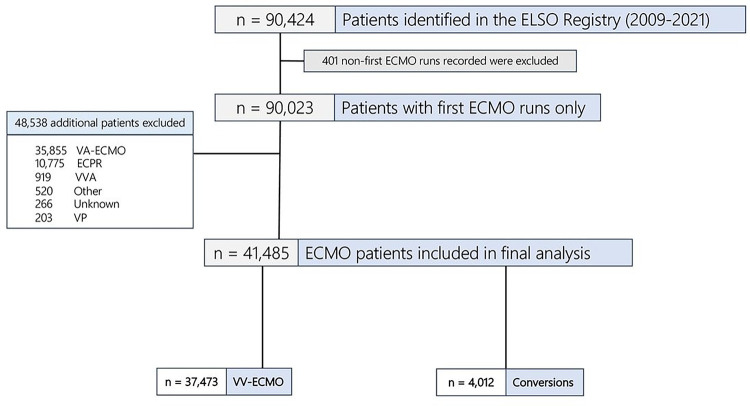
Creation of VV-ECMO and Conversion cohorts from the ELSO Registry (2009–2021). ECMO = extracorporeal membrane oxygenation, VA = venoarterial, VV = venovenous, Conversion = VA → VV or VV → VA, ECPR = extracorporeal cardiopulmonary resuscitation, VVA = venovenoarterial, Other = mode not defined, VP = venopulmonary.

In 37,473 total VV-ECMO patients, 2,644 (7.1%) experienced ABI (Fig. 2). The median age of the cohort was 48.1 years (IQR: 35.9–58.5 years) and 63% (n = 23,650) were male. The median duration of ECMO support was 9.9 days (4.0–20.3 days). VV-ECMO patients with ABI were more likely to be Asian and Hispanic, more likely to have pre-ECMO vasopressor infusions, and had higher ECMO pump flow rates at 4 and 24 hours of ECMO support than those without ABI (Table 1).

### Model Performance in VV-ECMO

The model obtained an AUC-ROC of 0.67 for predicting ABI (Table 2) with an accuracy of 79%. The true positive rate, true negative rate, false positive rate, and false negative rate were 39%, 84%, 17%, and 61%, respectively. The PPV and NPV were 15% and 95%, respectively. The precision, recall, and F1 values were 0.15, 0.39, and 0.21, respectively. The Brier score was 0.167.

For CNS ischemia, the model obtained an AUC-ROC of 0.63 (Table 2) with an accuracy of 94%. The true positive rate, true negative rate, false positive rate, and false negative rate were 15%, 95%, 15%, and 85%, respectively. The PPV and NPV were 5% and 99%, respectively. The precision, recall, and F1 values were 0.048, 0.26, and 0.07, respectively. The Brier score was 0.06.

For ICH, the model obtained an AUC-ROC of 0.70 (Table 2) with an accuracy of 89%. The true positive rate, true negative rate, false positive rate, and false negative rate were 28%, 92%, 8%, and 72%, respectively. The PPV and NPV were 13% and 97%, respectively. The precision, recall, and F1 values were 0.13, 0.28, and 0.18, respectively. The Brier score was 0.177.

### Feature Importance Scores in VV-ECMO

**Figure 4:**
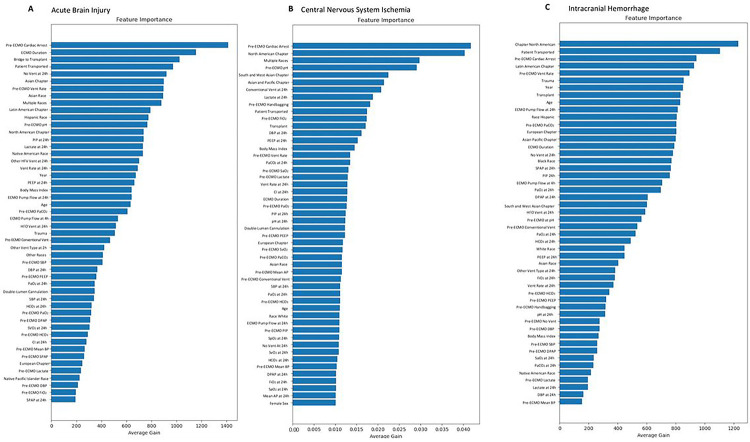
Most important features for each neurological outcome: **A)** acute brain injury, **B)** central nervous system ischemia, and **C)** intracranial hemorrhage in VV-ECMO patients. AP: airway pressure. BP: systolic blood pressure. CI: cardiac index. DBP: diastolic blood pressure. DPAP: diastolic pulmonary arterial pressure. ECMO: extracorporeal membrane oxygenation. HFV: high frequency ventilator. PEEP: positive-end expiratory pressure. PIP: peak inspiratory pressure. SvO_2_: mixed venous oxygen saturation. SPAP: systolic pulmonary arterial pressure. Vent: ventilator.

We identified the top 3 most important features by calculating average gains in Feature Importance Scores and presented the other significant variables (Fig. 4). The top 3 features for ABI prediction were pre-ECMO cardiac arrest, ECMO duration, and a bridge to transplant as an ECMO indication (Fig. 4A and **Supplemental Fig. 1A**). The prevalence of ABI was higher in pre-ECMO cardiac arrest patients versus those without cardiac arrest (13% vs 6.6%, p < 0.001). The median ECMO duration time for those with ABI was shorter versus those without ABI (9.2 versus 9.9 days, p = 0.01). The prevalence of ABI in those with an indication for bridge to transplantation was lower versus those without an indication for bridge to transplantation (3.6% versus 7.3%, p < 0.001). For predicting CNS ischemia, the top 3 features were pre-ECMO cardiac arrest, being supported on ECMO at a North American ELSO center, and patients who were of multiple races/ethnicities (e.g., both White and Black race/ethnicity) (Fig. 4B and **Supplemental Fig. 1B**). The prevalence of CNS ischemia was higher in pre-ECMO cardiac arrest patients versus those without pre-ECMO cardiac arrest (4.4% versus 1.4%, p < 0.001). The prevalence of CNS ischemia was higher in patients supported on ECMO at a North American ELSO Center versus those not supported on ECMO at a North American ELSO Center (2% versus 1.3%, p < 0.001). The prevalence of CNS ischemia was lower in patients of multiple races/ethnicities versus those of single race/ethnicity (1% versus 1.7%, p = 0.017). For predicting ICH, the top 3 variables were being supported on ECMO at a North American ELSO Center, being transported to another ECMO center while on ECMO support, and pre-ECMO cardiac arrest (Fig. 4C and **Supplemental Fig. 1c**). The prevalence of ICH was lower in patients supported on ECMO at a North American ELSO Center versus those not supported on ECMO at a North American ELSO Center (4% vs 4.6%, p = 0.013). The prevalence of ICH was not different between patients being transported to another ECMO center while on ECMO support versus those not being transported to another ECMO center while on ECMO support (5.5% versus 5.4%, p = 0.80). The prevalence of ICH was not different between pre-ECMO cardiac arrest patients versus those without pre-ECMO cardiac arrest patients (3.6% versus 4.3%, p = 0.065).

### Supplementary analysis of ECMO patients undergoing modality change

In 4,012 patients who were converted from one ECMO modality to another (“Conversions”), 466 (11.6%) experienced ABI (**Supplemental Fig. 2**). Among this cohort, 2,335 were converted from VA-ECMO to VV-ECMO and 1,677 were converted from VV-ECMO to VA-ECMO. The median age of the entire cohort that was converted from one modality to another was 53 years (IQR: 39.2–62.4 years) and 65% (n = 2,618) were male. The median duration of ECMO support was 9.9 days (4.9–20.3 days). ECMO patients who were converted from one modality to another with ABI were younger, were cannulated for longer, and had lower pre-ECMO pH and PaCO_2_ than those without ABI (**Supplemental Table 2**).

For ABI, the model obtained an AUC-ROC of 0.58 with an accuracy of 58% (**Supplemental Table 3**). For CNS ischemia, the AUC-ROC was 0.57 with an accuracy of 75% (**Supplemental Table 3**). For ICH, the AUC-ROC was 0.63 with an accuracy of 80% (**Supplemental Table 3**). The most important features for predicting ABI, CNS ischemia, and ICH in Conversion patients are depicted in **Supplemental Figs. 2 and 3.**

## Discussion

This is the first study to apply tree-based ML to predict ABI in adult respiratory failure VV-ECMO patients using a large, international multicenter database (the ELSO Registry) over a substantial period encapsulating two respiratory virus pandemics (2009–2021). ML predicted ABI with an AUC-ROC of 0.67 and we identified pre-ECMO cardiac arrest important clinical risk factor for ABI while longer duration of ECMO support and use of ECMO as a bridge to transplantation were associated with lower risk of ABI.

### Novelty of ML and ELSO Registry

Theoretically, ML allows for the appropriate analysis of exceedingly large datasets, improving efficiency and accuracy in prediction as the model develops more experience.^3,4^ ML also facilitates an environment to recognize new patterns and integrate data in a fashion that humans may be unable to achieve.^3^ The ELSO Registry allows us to generalize our results to a global population across multiple continents (North America, Europe, Asia, Pacific Islands, Latin America, Southwest Asia, and Africa) with an eclectic cohort of respiratory failure patients undergoing VV-ECMO support from various ELSO centers. This allows us to improve upon various studies in ECMO populations that used ML that are 1) from a single center; 2) small sample size; and/or 3) capture only one specific ECMO indication.^8–10^ Our study captured patients from over a decade, which may further contribute to the generalizability and real-world validity of our model. Furthermore, while a previous ELSO Registry study of 23,182 VA-ECMO patients demonstrated ML was able to predict survival with an AUC-ROC of 0.80,^5^ it should be noted that assessing mortality is a less complex outcome to discern than ABI, which requires a central adjudication and standardization of clinical practice, a limitation of the ELSO Registry data. Accordingly, ABI is likely underestimated in the ELSO Registry. Therefore, their good performance using ML to predict mortality (AUC-ROC = 0.80) in VA-ECMO and the current suboptimal performance in predicting ABI (AUC-ROC = 0.67) in VV-ECMO may not be surprising.

### VV-ECMO risk factors for ABI

We identified cardiac arrest 6 hours before ECMO support as the most important risk factor for ABI while longer duration of ECMO support and patients receiving ECMO support as a bridge to transplantation were the most important features associated with lower risk of ABI. While cardiac arrest is a known risk factor,^11,12^ current literature on the association between ECMO duration and ABI is lacking. The lower risk of ABI with ECMO duration may reflect selection bias of the subset of ECMO patients who underwent the decision to limit life-sustaining therapies in the setting of severe ABI or those patients who died directly from ABI and thus observed a shorter length of ECMO support. We uniquely identified ECMO patients bridged to transplant as a factor associated with lower risk of ABI (ABI prevalence was 3.6% versus 7.3% in patients indicated for bridge to transplant versus patients who were not), which may reflect a less severity of illness in this population. Finally, patients cannulated at a North American ELSO center were at higher risk of CNS ischemia and ICH versus non-North American ELSO centers. This finding may reflect the higher volume of ECMO cases in North America relative to other continents and a higher potential to cannulate overall sicker patients or an increased ability for North American ELSO centers to successfully detect ABI compared to ELSO centers across the world. This result may also reflect different practices for detecting ABI across different continents, which warrants further investigation.

### Need for standardized neuromonitoring

The prevalence of ABI in both VV-ECMO and Conversion cohorts was relatively low in the ELSO Registry, which likely contributed to the modest performance of our ML models to predict ABI. This notion may be supported by the corresponding low PPV but high NPV for ABI and its subtypes in both cohorts. Although the ELSO Registry comprises the largest cohort of ECMO patients available, data gathered from hundreds of different ECMO centers results in a heterogeneous cohort, especially with the methodology used to detect ABI varying across centers. In a prior study, noninvasive standardized neuromonitoring detected ABI in up to 33% of VA-ECMO patients,^13^ but this methodology has not been implemented by all ECMO centers. Centers implementing standardized neuromonitoring has been shown to detect ABI at a higher prevalence: 10%^14^ and 16%^15^ in VV-ECMO patients. This is supported by a single-center pediatric ECMO study (68 VV-ECMO and 106 VA-ECMO patients) demonstrating a high prevalence of ABI (51%) where standardized neuromonitoring with neuroimaging protocol was implemented.^16^ This study also used artificial intelligence to predict ABI primarily in VA-ECMO patients and demonstrated a better AUC-ROC (0.76)^16^ versus our AUC-ROC with the ELSO Registry (0.67 in VV-ECMO patients).

### Limitations

Our study has several limitations. First, our study was retrospective and observational in nature, so causation effects could not be assessed. Second, the prevalence of ABI was low within the ELSO Registry, likely due to lack of noninvasive neurological monitoring protocols standardized across all ECMO centers. Third, due to high heterogeneity in the data, we limited our analysis to which variables were most essential overall using Feature Importance Scores. Nevertheless, we were able to identify novel and highly important features for ABI that prior regression modeling was unable to perceive in the ELSO Registry and we determined the direction of these features by looking at prevalence of ABI in the raw data. Fourth, as pre-ECMO and on-ECMO hemodynamics and ABGs were only collected at a singular time point, we were unable to assess multiple time points for these important laboratory values in our ML models. However, it is unclear if these laboratory values would change substantially throughout the ECMO run or in the hours prior to cannulation. Fifth, anticoagulation is a known risk factor for ICH in VV-ECMO patients, but we could not capture this feature in the ELSO Registry. Finally, we did not externally validate our findings with an independent dataset, which is warranted in the future with granular data.

### Interpretation

This is the first study to use ML to predict ABI in a large cohort of VV-ECMO patients. However, its performance was sub-optimal due to the low reported prevalence of ABI from ECMO centers globally and lack of data granularity of the ELSO Registry. We identified cardiac arrest 6 hours prior to ECMO cannulation as the most important risk factor for ABI while longer length of ECMO support and ECMO as a bridge to transplantation as an indication for ECMO were associated with lower risk of ABI in VV-ECMO patients.

## Figures and Tables

**Figure 3:**
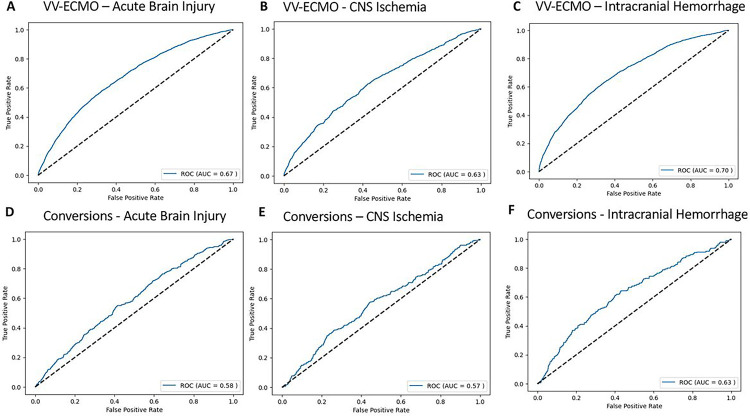
Receiver-operating characteristic curves (ROC) for predicting area under the receiver-operating characteristic curves (AUC) **A)** acute brain injury, **B)** central nervous system (CNS) ischemia, and **C)** intracranial hemorrhage in venovenous extracorporeal membrane oxygenation (VV-ECMO) and for predicting **D)** acute brain injury, **E)** CNS ischemia, and **F)** intracranial hemorrhage in “Conversion” patients.

**Table 1:** Baseline characteristics and clinical variables of venovenous extracorporeal membrane oxygenation patients stratified by presence of ABI.

	Total VV-ECMO (n = 37,437)	ABI (n = 2,644, 7%)	No ABI (n = 34,829, 93%)	P-value
**Demographics**				
Age (years)	48.1 (35.9-58.5)	48.1 (35.8-58.5)	48.7 (32.3-57.8)	0.43
Male sex	23,650 (63%)	1,672 (63%)	21,979 (63%)	0.98
Body Mass Index, kg/m^2^	30.1 (25.6-36.1)	28.4 (24.5-33.1)	30.11 (25.5-356.2)	0.06
**Race/ethnicity**				**< 0.001**
Asian	3,926 (10%)	319 (12%)	3,608 (10%)	
Black	4,151 (11%)	247 (9%)	3,905 (11%)	
Hispanic	3,905 (10%)	364 (14%)	3,673 (11%)	
White	19,812 (53%)	1,321 (50%)	18,492 (53%)	
Others	5,643 (15%)	393 (15%)	5,151 (15%)	
**Year ECLS**				
2009	357 (1%)	29 (1%)	328 (1%)	**< 0.001**
2010	410 (1%)	22 (1%)	388 (1%)	
2011	557 (1%)	43 (2%)	514 (1%)	
2012	836 (2%)	54 (2%)	782 (2%)	
2013	1,304 (3%)	86 (3%)	1,218 (3%)	
2014	1,777 (5%)	138 (5%)	1,639 (5%)	
2015	2,019 (5%)	140 (5%)	1,879 (5%)	
2016	2,773 (7%)	178 (7%)	2,595 (7%)	
2017	3,106 (8%)	150 (6%)	2,956 (8%)	
2018	3,693 (10%)	246 (9%)	3,447 (10%)	
2019	4,447 (12%)	267 (10%)	4,180 (12%)	
2020	7,716 (21%)	611 (23%)	7,105 (20%)	
2021	8,478 (23%)	680 (21%)	7,798 (22%)	
**Past medical history**				
Diabetes	2,738 (7%)	208 (8%)	2,530 (7%)	0.09
Hypertension	3,415 (9%)	277 (10%)	3,138 (9%)	0.09
Atrial fibrillation	1,581 (4%)	115 (4%)	1,466 (4%)	0.94
Cardiomyopathy	461 (1%)	29 (1%)	432 (1%)	0.64
COPD	1,583 (4%)	111 (4%)	1,472 (4%)	0.74
**Pre-ECMO support**				
Additional temporary mechanical circulatory support	899 (2%)	57 (2%)	842 (2%)	0.40
Vasopressor infusions	19,439 (52%)	1619 (61%)	17,820 (51%)	**< 0.001**
Inotrope infusions	2,203 (6%)	158 (6%)	2,045 (6%)	0.83
**Pre-ECMO blood pressure variables**				
Systolic blood pressure (mm Hg)	110 (95–128)	110 (70–127)	110 (95–128)	0.27
Diastolic blood pressure (mm Hg)	60 (51–70)	59 (50–68)	60 (51–70)	**< 0.001**
Mean blood pressure (mm Hg)	75 (65–86)	75 (65–85)	75 (65–86)	**0.03**
Pulse pressure (mm Hg)	50 (39–63)	50 (40–64)	50 (39–63)	0.16
Mean arterial pressure (mm Hg)	22 (18–26)	22 (19–26)	22 (18–26)	**0.002**
**Pre-ECMO ABG**				
pH	7.26 (7.17-7.35)	7.23 (7.13-7.32)	7.27 (7.17-7.35)	**< 0.001**
HCO_3_- (mEq/L)	25.90 (21.6-31)	26 (21.1–31)	25.9 (21.6-30.9)	0.91
PaO_2_ (mm Hg)	65 (53–81)	63 (51–78)	65 (53–82)	**< 0.001**
PaCO_2_ (mm Hg)	58.5 (33.80-74.5)	62.2 (49.5–80)	58 (46.6–74)	**< 0.001**
Lactate (mmol/L)	1.2 (1.8–3.1)	2 (1.3–4)	1.8 (1.2–3.1)	**< 0.001**
SpO_2_ (%)	90 (85–95)	90 (83–94)	90 (85–95)	**0.002**
SaO_2_ (%)	90 (83–94)	89 (81–94)	90 (83–94)	**< 0.001**
**On-ECMO blood pressure variables**				
Systolic blood pressure (mm Hg)	115 (103–129)	115 (103–129)	115 (103–128)	0.38
Diastolic blood pressure (mm Hg)	60 (53–68)	60 (53–68)	60 (53–67)	0.59
Mean blood pressure (mm Hg)	76 (70–85)	76 (69–86)	76 (70–85)	0.64
Pulse pressure (mm Hg)	55 (45–66)	5 (45–67)	55 (45–66)	0.14
Mean arterial pressure (mm Hg)	15 (12–18)	15 (13–19)	15 (12–18)	0.13
**On-ECMO ABG**				
pH	7.4 (7.36-7.44)	7.4 (7.35-7.44)	7.4 (7.36-7.44)	**0.03**
HCO_3_- (mEq/L)	26 (23–30)	26 (22.5–30)	26 (23–30)	0.28
PaO_2_ (mm Hg)	78 (64.5–103)	75 (62–99)	78 (65–103)	**< 0.001**
PaCO_2_ (mm Hg)	42.3 (37–48.2)	42 (37–48.5)	42.3 (37.1-48.1)	0.38
Lactate (mmol/L)	1.5 (1.1 –2.3)	1.6 (1.2–2.5)	1.5 (1.1 –2.3)	**< 0.001**
SpO_2_ (%)	96 (92–98)	95 (92–98)	96 (92–98)	**0.006**
SaO2 (%)	95 (92–98)	95 (92–97)	95 (92–98)	**0.007**
**ΔPaCO_2_**	−15 (−30 to −4)	−19 (−35.2 to −6.25)	−15 (−30 to −4)	**< 0.001**
**Pump flow rate (4 hours, L/min)**	4.05 (3.5–4.6)	4.05 (3.5–4.6)	4.05 (3.5–4.6)	**< 0.001**
**Pump flow rate (24 hours, L/min)**	4.1 (3.5–4.7)	4.17 (3.634.8)	4.1 (3.5–4.7)	**< 0.001**
**Days on ECMO support**	9.92 (4.88-20.3)	9.17 (3.83-21.6)	9.92 (4.92-20.2)	**0.01**
**Neurological complications on-ECMO**				
*Composite ABI*				
*Composite Ischemia*	610 (2%)	610 (23%)	0 (0%)	**< 0.001**
Hypoxic-ischemic brain injury	139 (1%)	139 (5%)	0 (0%)	**< 0.001**
Ischemic stroke	478 (1%)	478 (18%)	0 (0%)	**< 0.001**
*Composite ICH*	1,591 (4%)	1,591 (60%)	0 (0%)	**< 0.001**
Intra/extra parenchymal hemorrhage	745 (2%)	745 (28%)	0 (0%)	**< 0.001**
Intraventricular hemorrhage	306 (1%)	306 (12%)	0 (0%)	**< 0.001**
Brain death	462 (1%)	462 (17%)	0 (0%)	**< 0.001**
Neurosurgical intervention	37 (1%)	37 (1%)	0 (0%)	**< 0.001**
Seizures confirmed by EEG	107 (1%)	107 (4%)	0 (0%)	**< 0.001**
Seizures clinically determined	249 (1%)	249 (9%)	0 (0%)	**< 0.001**
**Other complications on-ECMO**				
ECMO circuit mechanical failure	9,112 (24%)	688 (26%)	8,424 (24%)	**0.03**
Renal replacement theory	9,772 (26%)	981 (37%)	8,791 (25%)	**< 0.001**
Hemolysis	1,851 (5%)	193 (7%)	1,658 (5%)	**< 0.001**
Cardiac arrhythmia	2,909 (8%)	298 (11%)	2,611 (7%)	**< 0.001**
Gastrointestinal hemorrhage	2,036 (5%)	200 (8%)	1,836 (5%)	**< 0.001**
**Outcomes**				
In-hospital mortality	15,074 (40%)	2,088 (79%)	12,986 (37%)	**< 0.001**

Δ = delta. ABG: arterial blood gases. ABI: acute brain injury. ICH: intracranial hemorrhage. VA-ECMO: venoarterial extracorporeal membrane oxygenation.

**Table 2:** Model performance in venovenous extracorporeal membrane oxygenation patients for predicting acute brain injury, central nervous system ischemia, and intracranial hemorrhage.

	AUC- ROC	Acc	TPR	TNR	FPR	FNR	PPV	NPV	Precision	Recall	F1	Brier Score
ABI	0.67	79%	39%	84%	17%	61%	15%	95%	0.15	0.39	0.21	0.167
CNS Ischemia	0.63	94%	15%	95%	5%	85%	5%	99%	0.048	0.16	0.07	0.06
ICH	0.70	89%	28%	92%	8%	72%	13%	97%	0.13	0.28	0.18	0.177

AUC-ROC: area under the receiver-operating characteristic curve. Acc: Accuracy. TPR: True Positive Rate. TNR: True Negative Rate. FPR: False Positive Rate. FNR: False Negative Rate. PPV: Positive Predictive Value. NPV: Negative Predictive Value. ABI: acute brain injury. CNS: central nervous system. ICH: intracranial hemorrhage.

## Take Home Points

### Study Question:

Can machine learning be used to accurately predict acute brain injury (ABI) and identify modifiable factors in venovenous-extracorporeal membrane oxygenation (VV-ECMO) patients?

### Results:

Of 37,473 VV-ECMO patients from 676 ECMO centers from the Extracorporeal Life Support Organization Registry, 2,644 (7.1%) experienced ABI; machine learning predicted ABI with an area under the receiver-operating characteristics curve of 0.67 and identified that the most important associations with ABI were pre-ECMO cardiac arrest (higher risk of ABI) and ECMO duration and ECMO as a bridge to transplantation as an indication (both lower risk of ABI).

### Interpretation:

This is the first study using machine learning to predict ABI in a large cohort of VV-ECMO patients; we found machine learning suboptimally predicted ABI but still identified pre-ECMO cardiac arrest as the most important risk factor for ABI while bridge to transplantation as an indication for ECMO were associated with lower risk of ABI.

## Abbreviations

ABG: arterial blood gas
ABI: acute brain injury
AUC-ROC: area under the receiver-operating characteristic curve
BMI: body mass index
CI: confidence interval
CNS: central nervous system
DBP: diastolic blood pressure
DPAP: diastolic pulmonary arterial pressure
ECMO: extracorporeal membrane oxygenation
ELSO: Extracorporeal Life Support Organization
ICH: intracranial hemorrhage
IQR: interquartile range
LOOCV: leave-one-out-cross-validation
ML: machine learning
MPAP: mean positive airway pressure
PaCO_2_: partial pressure of carbon dioxide
PaO_2_: partial pressure of oxygen
PCWP: positive capillary wedge pressure
PEEP: positive end-expiratory pressure
PIP: positive inspiratory pressure
PP: pulse pressure
SBP: systolic blood pressure
SD: standard deviation
SHAP: Shapley Additive Explanations
SPAP: systolic pulmonary arterial pressure
SvO_2_: venous oxygen saturation
VA: venoarterial
VV: venovenous
